# Supplementary material for: Distinct contributions of functional and deep neural network features to representational similarity of scenes in human brain and behavior
Source: eLife. 2018 Mar 7;7:e32962. doi: 10.7554/eLife.32962 (PMC5860866; doi:10.7554/eLife.32962)
Supplement: Figure 1—source data 1. [file elife-32962-fig1-data1.zip › README.rtf]

#### Supplementary Data File 1 Groen et al. ####The Supplementary Data is a matlab readable .mat struct with the following fields:GROENETAL.data:This field is further subdivided in Experiment 1 and 2, which contain: * single-subject RDMs for behavior (Exp 1 only). Dimensions: 20 (subjects) x 435 (vectorized lower triangle)* single-subject RDMs for scene-selective ROIs. Dimensions: 3 (ROIs) x 20 (subjects) x 435.* an index for stimset (1 or 2) and task type (fix vs naming) for each subject. Dimensions: 20 x 1 * subject IDs (such that participants that performed both Exp 1 and 2 can be cross-matched). Dimensions 20 x 1.GROENETAL.modelsThis field contains RDMs for:* the three a priori models tested in the study (function, dnnfc7, objects). Dimensions: 1 x 435.* the behavior for the online categorization task reported in Greene et al. (2016). Dimensions: 1 x 435.* each layer of the Places and Refnet trained DNNs. Dimensions: 8 (layers) x 435.GROENETAL.stimuli This field contains: * the stimuli themselves, subdivided in the two stimulus sets containing 120 stimuli each.* an index indicating which image belongs to which category. Dimensions: 120 x 1* a list of the category names. Dimensions: 30 x 1. 
