# Supplementary material for: Distinct contributions of functional and deep neural network features to representational similarity of scenes in human brain and behavior
Source: eLife. 2018 Mar 7;7:e32962. doi: 10.7554/eLife.32962 (PMC5860866; doi:10.7554/eLife.32962)
Supplement: Transparent reporting form [file elife-32962-transrepform.docx]

***eLife’s* transparent reporting form**

We encourage authors to provide detailed information *within their submission* to facilitate the interpretation and replication of experiments. Authors can upload supporting documentation to indicate the use of appropriate reporting guidelines for health-related research (see [EQUATOR Network](http://www.equator-network.org/%20)), life science research (see the [BioSharing Information Resource](https://biosharing.org/)), or the [ARRIVE guidelines](http://www.plosbiology.org/article/info:doi/10.1371/journal.pbio.1000412) for reporting work involving animal research. Where applicable, authors should refer to any relevant reporting standards documents in this form.

If you have any questions, please consult our Journal Policies and/or contact us: [editorial@elifesciences.org](mailto:editorial@elifesciences.org).

**Sample-size estimation**

- You should state whether an appropriate sample size was computed when the study was being designed
- You should state the statistical method of sample size computation and any required assumptions
- If no explicit power analysis was used, you should describe how you decided what sample (replicate) size (number) to use

Please outline where this information can be found within the submission (e.g., sections or figure legends), or explain why this information doesn’t apply to your submission:

The sample size was determined based on prior fMRI studies employing representational similarity analyses (e.g., Kriegeskorte et al., 2008, Neuron; Kravitz et al., 2011, Journal of Neuroscience; Bonner et al., 2017, PNAS). For Experiment 1, a sample size of n = 20 was set beforehand. For Experiment 2, the sample size was reduced (n = 8) because the purpose of this experiment was to see if the same pattern of results would be obtained as in Experiment 1. This information is specified under *Results > Participants*.

**Replicates**

- You should report how often each experiment was performed
- You should include a definition of biological versus technical replication
- The data obtained should be provided and sufficient information should be provided to indicate the number of independent biological and/or technical replicates
- If you encountered any outliers, you should describe how these were handled
- Criteria for exclusion/inclusion of data should be clearly stated
- High-throughput sequence data should be uploaded before submission, with a private link for reviewers provided (these are available from both GEO and ArrayExpress)

Please outline where this information can be found within the submission (e.g., sections or figure legends), or explain why this information doesn’t apply to your submission:

Our study contains both biological (same experiment run on different samples) and technical (different experiment run on same samples) replicates.

In Experiment 1 two groups of subjects (each n = 10) participated under identical task instructions, but viewed different stimulus exemplars from the same scene categories, allowing for an internal biological replication within this Experiment. Similarly, in Experiment 2, again two groups of subjects saw the two stimulus sets (n = 3 vs. n = 5). We performed an explicit statistical test to assess similarity of the brain and behavioral measurements across these two stimulus sets. This test is described under *Methods > Direct reproducibility test of representational structure in behavior and fMRI*, and its outcomes are reported in several paragraphs of the *Results* section.

In addition, 4 participants from Experiment 1 also participated in Experiment 2, this time under a different task instruction as well as a different set of exemplars. This allowed for a test of the influence of task instruction. Distribution of sets and tasks are reported under *Methods > Participants* and *Methods > Stimuli & models*. The results of this test are reported in Figure 5D.

Criteria for inclusion were that participants had to complete the entire experimental protocol (fMRI scan and behavioral experiment). Beyond the sample reported in the manuscript, three additional subjects were scanned but behavioral data was either not obtained or lost. Four additional participants did not complete the scan session due to discomfort or technical difficulties.

In Experiment 1, one of the regions-of-interest (MPA) used for analysis of the fMRI data could only be identified at sub-threshold level in 6 subjects, and thus these subjects were excluded from analyses for this ROI. This information is reported under *Methods > fMRI statistical analysis: localizers*.

**Statistical reporting**

- Statistical analysis methods should be described and justified
- Raw data should be presented in figures whenever informative to do so (typically when N per group is less than 10)
- For each experiment, you should identify the statistical tests used, exact values of N, definitions of center, methods of multiple test correction, and dispersion and precision measures (e.g., mean, median, SD, SEM, confidence intervals; and, for the major substantive results, a measure of effect size (e.g., Pearson's r, Cohen's d)
- Report exact p-values wherever possible alongside the summary statistics and 95% confidence intervals. These should be reported for all key questions and not only when the p-value is less than 0.05.

Please outline where this information can be found within the submission (e.g., sections or figure legends), or explain why this information doesn’t apply to your submission:

The fMRI data was preprocessed using standard procedures using publicly available software (AFNI). Our behavioral and fMRI data were further analyzed using Representational Similarity Analysis (RSA), which allows for direct comparison behavioral, brain and computational domains by converting the data from each domain into a representational dissimilarity matrix (RDM) reflecting the similarity across stimulus conditions within each domain. The distance metrics used to construct RDMs in each domain are reported under *Methods > Behavioral data analysis* and *Methods > fMRI statistical analysis: event-related data*.

For our statistical tests comparing RDMs across domains, we generally followed directions suggested by Nili et al. (2014) A Toolbox for Representational Similarity Analysis. PLoS Comput Biol. Between-RDM analyses are described under *Methods > Model comparisons*. In brief, relations between RDMs are expressed in terms of correlation (Pearson’s *r*) or explained variance (*R^2^*). Significance of these relations at the group level was determined using both non-parametric across-subject tests (that support inference to the general population) as well as fixed-effects analyses, whereby RDMs were first averaged across before serving as inputs for reproducibility assessments and variance partitioning analysis. For across-subject tests, we report mean + SEM; for fixed-effects tests, we report mean + 95% confidence intervals (determined using permutation tests), in addition to exact p-values.

Figures 2-5 display data points for individual subjects in addition to the across-subject means. Exact values of n are reported for each across-subject test. For the behavioral and fMRI regions-of-interest analyses, False Discovery Rate (FDR) correction was applied to control for comparisons with and between multiple models within a single ROI. For the fMRI whole-brain analyses (described under *Methods > Searchlight analyses*), cluster-correction was performed using a threshold-free algorithm developed specifically for multi-voxel pattern analysis, implemented by Oosterhof NN et al. (2016) CoSMoMVPA: multi-modal multivariate pattern analysis of neuroimaging data in Matlab / GNU Octave. Front Neuroinform. 10:1–27

(For large datasets, or papers with a very large number of statistical tests, you may upload a single table file with tests, Ns, etc., with reference to sections in the manuscript.)

**Group allocation**

- Indicate how samples were allocated into experimental groups (in the case of clinical studies, please specify allocation to treatment method); if randomization was used, please also state if restricted randomization was applied
- Indicate if masking was used during group allocation, data collection and/or data analysis

Please outline where this information can be found within the submission (e.g., sections or figure legends), or explain why this information doesn’t apply to your submission:

The allocation of participants to the groups within Experiment 1 was random (subjects were assigned to specific stimulus sets alternately based on when they were recruited). The allocation of participants to the groups within Experiment 2 was initially based on the four participants that also participated in Experiment 1: they were assigned the stimulus set they had not yet seen in Experiment 1, and additional subjects were recruited to balance the sample in terms of stimulus sets collected. Distribution of sets and tasks are reported under *Methods > Participants and Methods > Stimuli & models*.

**Additional data files (“source data”)**

- We encourage you to upload relevant additional data files, such as numerical data that are represented as a graph in a figure, or as a summary table
- Where provided, these should be in the most useful format, and they can be uploaded as “Source data” files linked to a main figure or table
- Include model definition files including the full list of parameters used
- Include code used for data analysis (e.g., R, MatLab)
- Avoid stating that data files are “available upon request”

Please indicate the figures or tables for which source data files have been provided:

To facilitate replicability and to allow for potential comparisons of additional or future models against the behavioral and fMRI data reported in this study, the numerical data making up the RDMs for fMRI, behavior, and computational models (i.e. the source data for the analyses reported in Figures 2-7) are provided as Supplementary Data.
